# Supplementary material for: Travel surveillance uncovers dengue virus dynamics and introductions in the Caribbean
Source: medRxiv. 2023 Nov 12:2023.11.11.23298412. Preprint. [Version 1] doi: 10.1101/2023.11.11.23298412 (PMC10659465; doi:10.1101/2023.11.11.23298412)
Supplement: Supplement 1 [file NIHPP2023.11.11.23298412v1-supplement-1.pdf]

medRxiv preprint doi: https://doi.org/10.1101/2023.11.11.23298412; this version posted November 12, 2023. The copyright holder for this preprint (which was not certified by peer review) is the author/funder, who has granted medRxiv a license to display the preprint in perpetuity. This article is a US Government work. It is not subject to copyright under 17 USC 105 and is also made available for use under a CC0 license.

Supplement

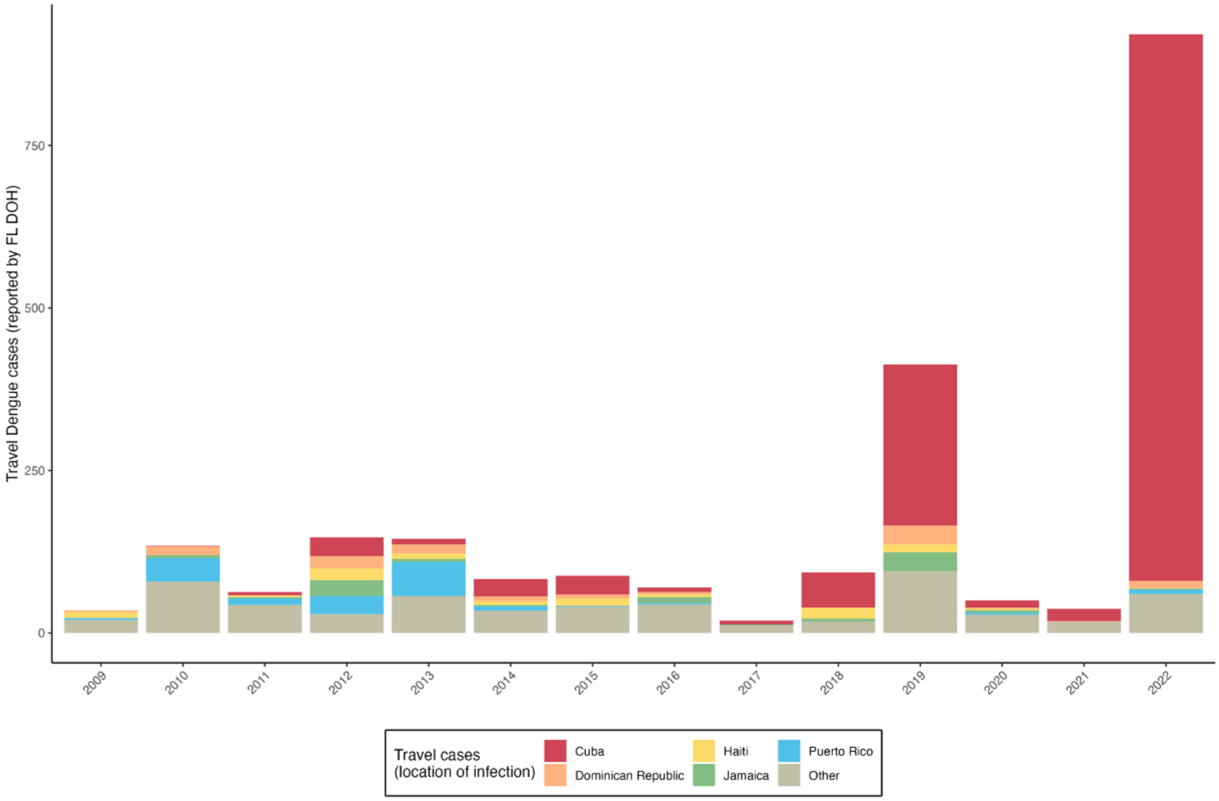

Figure S1. Total number of travel-associated dengue cases reported in Florida from 2009 to 2022. Travel-associated dengue cases per year for the top five locations of travel/exposure shown in **Figure 1**.

medRxiv preprint doi: https://doi.org/10.1101/2023.11.11.23298412; this version posted November 12, 2023. The copyright holder for this preprint (which was not certified by peer review) is the author/funder, who has granted medRxiv a license to display the preprint in perpetuity. This article is a US Government work. It is not subject to copyright under 17 USC 105 and is also made available for use under a CC0 license.

Travel cases from locations outside the top five were combined into the "Other" category. Overall, there were 1283 travel cases from Cuba (57.4%), 106 cases from Dominican Republic (4.7%), 85 cases from Haiti (3.8%), 90 cases from Jamaica (4.0%) and 149 cases from Puerto Rico (6.5%).

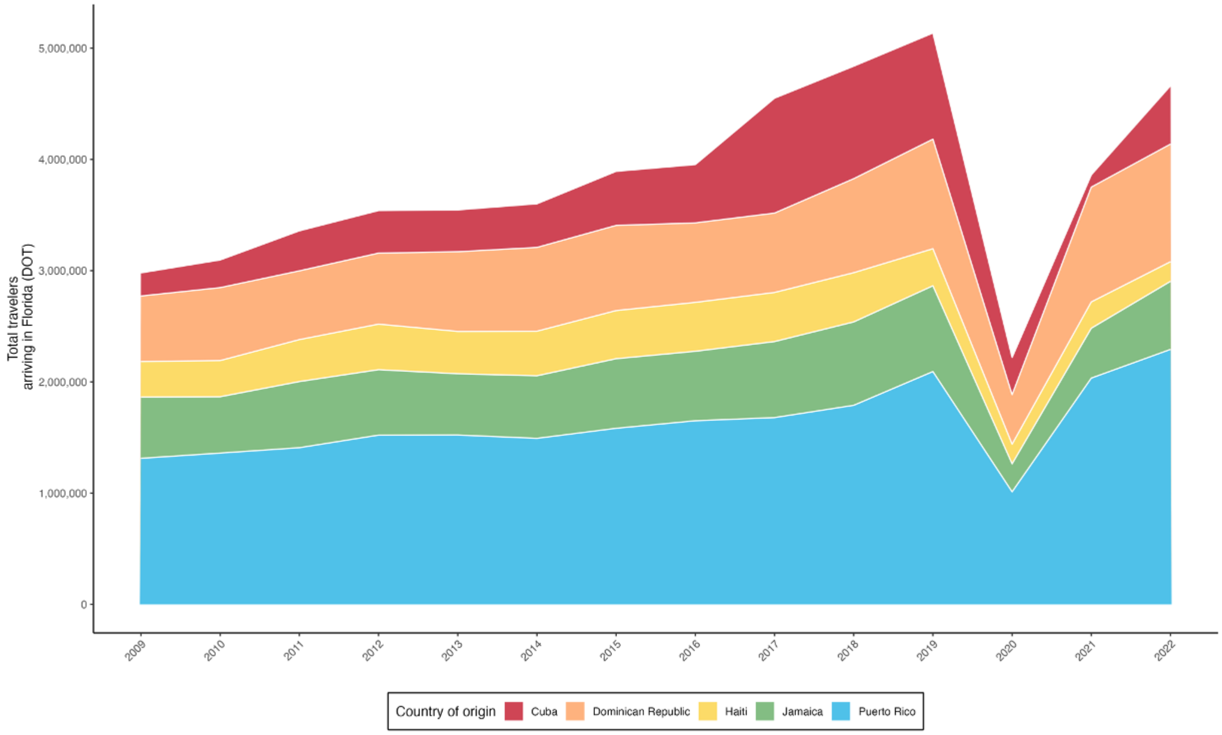

Figure S2. Total number of air passenger journeys from Caribbean countries and territories into Florida from 2009 to 2022. Total number of air passenger journeys reported by the United States Department of Transportation (DOT) from the top five locations of travel/exposure shown in **Figure 1**. These data were used to calculate the dengue travel infection rates shown in **Figure 2** and used in the model in **Figure 3**. Air travel was low in 2020 due to COVID-19 pandemic travel restrictions.

medRxiv preprint doi: https://doi.org/10.1101/2023.11.11.23298412; this version posted November 12, 2023. The copyright holder for this preprint (which was not certified by peer review) is the author/funder, who has granted medRxiv a license to display the preprint in perpetuity. This article is a US Government work. It is not subject to copyright under 17 USC 105 and is also made available for use under a CC0 license.

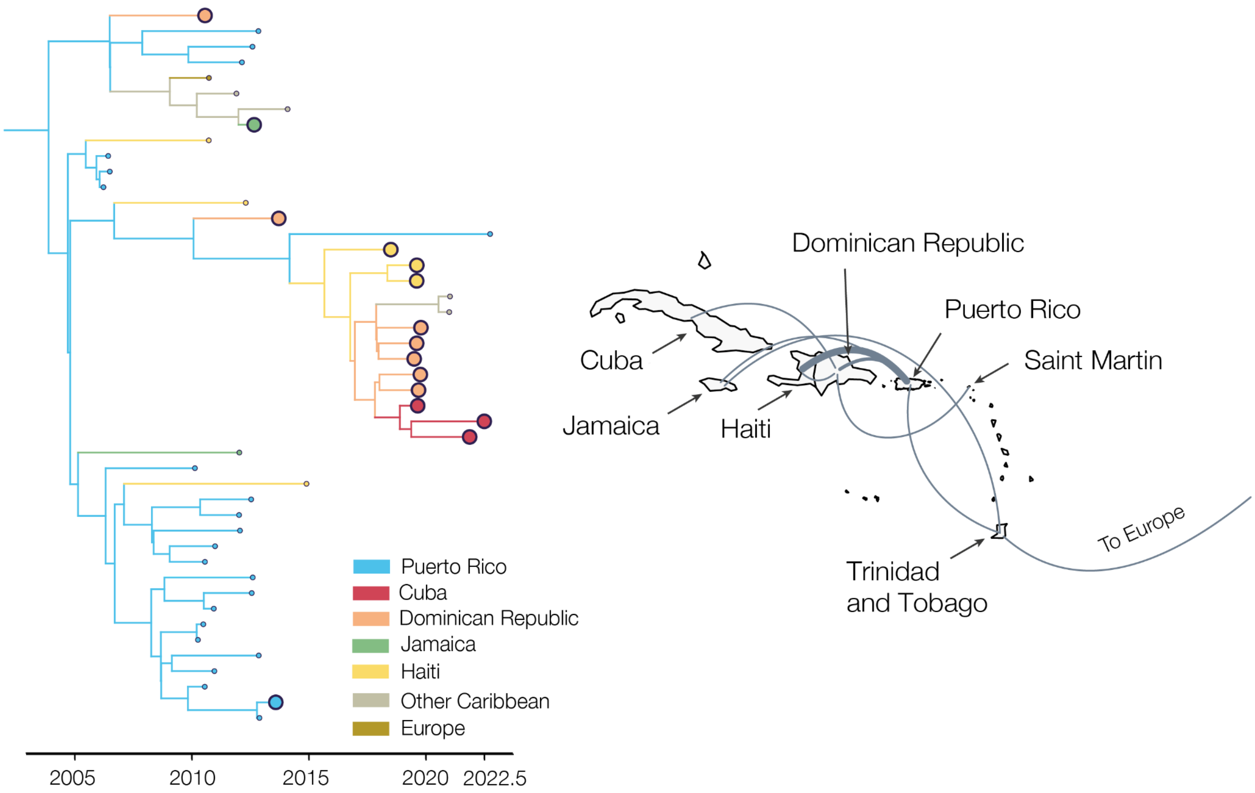

Figure S3. Within-Caribbean spread of a DENV-2 clade.

Time-scaled subtree of DENV-2 colored by inferred and sampled locations. Shows transitions from Puerto Rico, mostly to the Dominican Republic, and further onto Haiti and Cuba. Map shows the region, with "Other Caribbean" movements going directly to the islands in question.

medRxiv preprint doi: https://doi.org/10.1101/2023.11.11.23298412; this version posted November 12, 2023. The copyright holder for this preprint (which was not certified by peer review) is the author/funder, who has granted medRxiv a license to display the preprint in perpetuity. This article is a US Government work. It is not subject to copyright under 17 USC 105 and is also made available for use under a CC0 license.

Table S1. Total travel-associated dengue cases reported by the FDOH from 2009-2022, broken down by region, country or territory, year and serotype. Serotype data was not available for all cases. For Cuba in 2019 and 2022, both the total number of cases and the number of cases first identified through syndromic surveillance are included.

Travel-associated dengue cases

Reported by Florida Department of Health

Country of Likely Exposure	Year	DENV-1	DENV-2	DENV-3	DENV-4	Mixed	Total Cases (syndromic)
**Caribbean**							
Antigua and Barbuda	2019	0	0	1	0	0	1
Antigua and Barbuda	2020	0	0	1	0	0	1
Aruba	2011	0	0	0	0	0	1
Bahamas	2020	1	0	0	0	0	1
Bahamas	2011	8	0	0	0	0	13
Barbados	2013	0	0	0	0	0	1
Belize	2019	0	2	0	0	0	2
Caribbean	2013	1	0	0	1	0	2
Caribbean	2014	0	0	0	0	0	1
Caribbean	2022	0	0	1	0	0	1
Cayman Islands	2010	0	0	0	0	0	1
Cuba	2013	1	2	1	2	0	9
Cuba	2014	3	5	2	1	0	27
Cuba	2020	6	1	1	0	0	11
Cuba	2021	8	5	1	0	1	19
Cuba	2019	18	192	0	0	3	248 (18)
Cuba	2022	43	91	550	49	7	848 (397)
Cuba	2010	0	0	0	0	0	1
Cuba	2011	0	0	0	2	0	5
Cuba	2012	0	0	1	17	1	29
Cuba	2015	0	5	11	0	1	29
Cuba	2016	0	0	3	2	0	7
Cuba	2017	0	6	0	0	0	6
Cuba	2018	0	41	0	0	0	54
Cuba/Bahamas	2014	0	1	0	0	0	1
Cuba/Jamaica	2019	0	0	1	0	0	1
Dominica	2013	0	0	0	1	0	1
Dominica	2019	0	0	2	0	0	3
Dominican Republic	2014	1	2	0	2	0	7
Dominican Republic	2015	1	2	0	0	0	6
Dominican Republic	2022	1	9	0	0	0	11
Dominican Republic	2010	2	1	0	0	0	13
Dominican Republic	2020	2	0	0	0	0	2
Dominican Republic	2013	6	0	0	0	0	14
Dominican Republic	2012	7	2	0	1	0	19
Dominican Republic	2019	24	1	0	0	0	29
Dominican Republic	2009	0	0	0	0	0	3
Dominican Republic	2011	0	1	0	0	0	1
Dominican Republic	2016	0	0	0	0	0	4
Dominican Republic	2021	0	1	0	0	0	1
[illegible]	[illegible]	[illegible]	[illegible]	[illegible]	[illegible]	[illegible]	[illegible]

medRxiv preprint doi: https://doi.org/10.1101/2023.11.11.23298412; this version posted November 12, 2023. The copyright holder for this preprint (which was not certified by peer review) is the author/funder, who has granted medRxiv a license to display the preprint in perpetuity. This article is a US Government work. It is not subject to copyright under 17 USC 105 and is also made available for use under a CC0 license.

Table S2 Number of sequences in phylogeographic discrete trait analysis by region and serotype obtained from GenBank and after down-sampling.

Region	DENV-1	DENV-2	DENV-3	DENV-4
Africa	43	78	16	2
Central America	84	109	47	9
Cuba	22	108	165	18
Dominican Republic	7	19	0	3
Eastern Mediterranean	8	30	9	1
Europe	6	2	0	0
Haiti	6	3	0	5
Jamaica	3	3	10	1
North America	1	5	0	0
Other Caribbean	19	12	11	2
Puerto Rico	55	199	108	45
South America	212	217	159	55
South East Asia	220	258	182	117
Western Pacific	409	363	132	87

medRxiv preprint doi: https://doi.org/10.1101/2023.11.11.23298412; this version posted November 12, 2023. The copyright holder for this preprint (which was not certified by peer review) is the author/funder, who has granted medRxiv a license to display the preprint in perpetuity. This article is a US Government work. It is not subject to copyright under 17 USC 105 and is also made available for use under a CC0 license.

Table S3 Number of sequences from travelers by country or territory and serotype sequenced in this study.

Country	DENV-1	DENV-2	DENV-3	DENV-4
Bolivia	2	0	0	1
Brazil	3	0	0	0
Cambodia	1	0	0	0
Colombia	1	1	1	0
Costa Rica	1	1	0	0
Cuba	21	88	96	14
Dominica	0	0	0	1
Dominican Republic	7	2	0	1
El Salvador	0	0	0	1
Guatemala	0	1	0	0
Haiti	3	1	0	1
Honduras	1	4	0	0
India	0	3	0	0
Jamaica	1	0	10	1
Mexico	2	3	0	0
Nicaragua	0	7	0	2
Paraguay	0	0	0	1
Philippines	0	1	0	0
Puerto Rico	1	2	0	4
Saint Lucia	0	0	1	0
Venezuela	2	2	0	0
